# Precision Temperature Control for the Laser Gyro Inertial Navigation System in Long-Endurance Marine Navigation

**DOI:** 10.3390/s21124119

**Published:** 2021-06-15

**Authors:** Zhenyu Xiong, Guo Wei, Chunfeng Gao, Xingwu Long

**Affiliations:** College of Advanced Interdisciplinary Studies, National University of Defense Technology, Changsha 410073, China; xiongzhenyu10@nudt.edu.cn (Z.X.); weiguo@nudt.edu.cn (G.W.); l18670346275@163.com (X.L.)

**Keywords:** thermal control, accelerometer, back propagation neural network, thermal analysis

## Abstract

In the Ring Laser Gyro Inertial Navigation System (RLG INS), the temperature characteristics of the accelerometer can directly influence the measurement results. In order to improve navigation accuracy in long-endurance marine navigation, the operating temperature of the accelerometer should be precisely controlled. Based on thermal studies on the accelerometer, temperature control precision should be better than 0.01 °C to achieve 1 × 10^−5^ m/s^2^ output accuracy of the accelerometer. However, this conclusion is obtained by approximate calculations and cannot be directly applied to different inertial navigation systems. In order to verify this thermal conclusion and broaden its application, the Back Propagation Neural Network (BP-NN) algorithm is adopted to validate the feasibility of temperature control in this paper. In addition, a multi-level temperature control system is also set up and carefully designed to support the validation and experiments under different conditions. Test results of the temperature control system prove that operating temperature variation can be reduced to 0.01 °C. Meanwhile, the standard deviation per hundred seconds of the accelerometer outputs, after temperature control, reaches 1 × 10^−5^ m/s^2^. Static altitude and navigation results were improved by 41.97% and 62.91%, respectively, with the precision temperature control system, which meets the long-endurance marine navigation requirements.

## 1. Introduction

The Ring Laser Gyro Inertial Navigation System (RLG INS) has been regarded as an indispensable system in the navigation field. The specific force measured by the accelerometer provides the whole system with acceleration information. Combining with angular rate increment information measured by the RLG, the location of carrier can be obtained by calculation [1]. In recent times, many application fields such as long-endurance marine navigation demand higher precision locating results from the RLG INS, thus it is necessary to optimize inertial sensors in order to improve navigation accuracy [2].

Performances of the RLG and the accelerometer can be improved through advances in manufacturing techniques, inner structural optimization and the usage of new materials. In addition, it is also effective to improve the accuracy of inertial sensors by reducing the impacts of temperature fluctuation [3]. Since the RLG is an optical sensor which is mainly made up of thermal-insensitive materials, the mechanical accelerometer is more easily influenced by temperature variation [4]. In recent times, temperature stabilization is considered important in numerous application fields, such as luminescence detection [5], semiconductor production [6], biological diagnostics [7], magneto-optical system [8] and optical system manufacturing [9]. Therefore, this paper aims at analyzing thermal influences on the accelerometer and verifying current temperature control theories.

Considering that the temperature variation can directly influence the accuracy of the accelerometer measurement, many factors such as the deformation of the quartz pendulous, the thermal conductivity of the frame and scale factors should be taken into consideration in order to eliminate thermal influences [10]. Theoretical analysis of the accelerometer was explained by D.H. Titterton and J.L. Weston [11]. The principle of the accelerometer was also discussed and error models were derived to analyze influenced factors.

Based on these analysis and models, there are commonly two methods adopted to solve temperature fluctuation problems: the former is temperature compensation while the latter is precise temperature control.

The temperature compensation method builds different simulated models to determine the relationships between the temperature and the outputs of the inertial sensors. Errors caused by temperature variations can be compensated when inertial sensors are well modeled [12]. Hong [13] and Dzhashitov [14] studied the temperature characteristics of inertial sensors. They developed temperature compensation models of different inertial sensors and proved that these models can effectively compensate for the bias of the inertial sensors. In addition, J.M. Gao [15] studied the relationships between the output voltage of the accelerometer and its operating temperature. Then static and dynamic temperature compensation models were established to reduce the temperature influences on the accelerometer measurement accuracy. 

However, when the application fields require higher navigation accuracy or the operating environment temperature varies greatly, the temperature compensation method might be disabled [16]. In order to solve this problem, a precise temperature control scheme is proposed. With extra thermal insulation structure and inner temperature control devices, the operating temperature of the inertial sensors, especially accelerometers, can be sustained at a proper point [17]. Disturbance from temperature variation can be reduced, while the output accuracy of the accelerometer can be improved to meet application demands. 

Lee studied and designed a temperature control system with a piezoresistor to reduce the temperature drift of the accelerometer [18]. In addition, Liu and Hu [19] also proposed a three-level temperature control system to isolate the temperature variation on a marine gravimeter. They modeled the gravimeter and subjected their control system to different temperature conditions. In addition, Li [20] and Cao [21] made an intensive study of the quartz flexible accelerometer in the RLG INS. They proposed detailed theoretical analysis, simulations and experiments to verify the precise temperature control scheme. Their temperature control accuracy reached ±0.02 °C, meeting the requirements of airborne gravity measurement.

These articles referenced above made achievements in analyzing the principle of the RLG INS and temperature impacts on the accelerometer. However, theoretical analysis on temperature control is partly obtained by approximated calculation and cannot be applied to different systems directly. In order to verify this analysis, this paper sets up simulated models and a multi-level precise temperature control system to achieve validation.

Considering that the accelerometer can be regarded as a nonlinear system, the Back Propagation Neural Network (BP-NN) algorithm is adopted to determine the relation of the model between the operating temperature of the accelerometer and its output acceleration [22]. The well-trained simulated model generated by the BP-NN algorithm is used to verify the theoretical thermal analysis and to provide guidelines for the design of the precise temperature control system.

After the verification of simulation by the BP-NN algorithm, a multi-level precise temperature control system is set up to further validate previous conclusions. This control system is tested under different conditions to evaluate its performance. Contrast experiments reflect that the temperature variation on the accelerometer is able to be limited to ±0.01 °C by the system and accuracy of the accelerometer has also been improved to 1 × 10^−5^ m/s^2^. Performance experiments verified the feasibility of the temperature control system in different situations. In conclusion, the attitude and static navigation experiments also prove that an improvement can be made to the measurement accuracy of the RLG INS.

## 2. Materials and Methods 

In this section, thermal impacts on the accelerometer are analyzed and temperature control accuracy is given. The BP-NN algorithm is adopted to validate the conclusion of thermal analysis. 

### 2.1. Theoretical Analysis on Inertial Navigation System and Accelerometer

It is defined that bold variables represent vectors and matrices, while non-bold variables represent scalars, in this text. The INS attitude error model can be derived as

(1) $$\dot{\phi }=\phi \times \omega_{in}^{n}+\delta \omega_{in}^{n}-C_{b}^{n}\delta \omega_{ib}^{b}$$

(2) $$\omega_{in}^{n}=\omega_{ie}^{n}+\omega_{en}^{n}=\left[\begin{array}{c} -\frac{V_{N}}{R_{M}+h}\\ \omega_{ie}\cos L+\frac{V_{E}}{R_{N}+h}\\ \omega_{ie}\sin L+\frac{V_{E}\tan L}{R_{N}+h} \end{array}\right]$$

where the i frame is the inertial coordinate system, the b frame is the carrier coordinate system and defined as the right-front-up, and the n frame is the navigation coordinate system and is defined as east-north-upward. ϕ is the misalignment attitude angle, $\omega_{in}^{n}$ is the angle rate of n frame relative to the i frame on the n frame, $C_{b}^{n}$ is the direction cosine matrix of the b frame relative to the n frame, $\delta \omega_{ib}^{b}$ is the angle rate error of the gyroscope output, $\omega_{ie}^{n}$ is the rotational angular rate of the earth on the n frame, $\omega_{en}^{n}$ is the is the angle rate of the n frame relative to the e frame on the n frame, L is the latitude, $V_{N}$ and $V_{E}$ are the velocity of the carriers on the north and east directions, respectively, and $R_{M}$ and $R_{N}$ are the radii of curvature in meridian and prime vertical, respectively. 

According to the specific force equation, the acceleration of the carrier on the n frame can be derived as

(3) $${\dot{v}}_{e}^{n}=C_{b}^{n}f^{b}-(2\omega_{ie}^{n}+\omega_{en}^{n})\times v_{e}^{n}+g^{n}$$

where ${\dot{v}}_{e}^{n}$ and $v_{e}^{n}$ are the acceleration and velocity, respectively, of the carrier, $f^{b}$ is the specific force generated by the accelerometer, and $g^{n}$ is the gravitational vector on the n frame.

Based on (3), the INS velocity error model is shown below

(4) $$\delta {\dot{v}}_{e}^{n}=f^{n}\times \phi +C_{b}^{n}\delta f^{b}-(2\omega_{ie}^{n}+\omega_{en}^{n})\times \delta v_{e}^{n}+v_{e}^{n}\times (2\delta \omega_{ie}^{n}+\delta \omega_{en}^{n})+\delta g^{n}$$

where $\delta {\dot{v}}_{e}^{n}$ is the acceleration error, $f^{n}$ is the specific force on the n frame, ϕ is the attitude error, $\delta f^{b}$ is the specific force error, $\delta v_{e}^{n}$ is the velocity error, $\delta \omega_{en}^{n}$ and $\delta \omega_{ie}^{n}$ are the errors of $\omega_{en}^{n}$ and $\omega_{ie}^{n}$, respectively, and $\delta g^{n}$ is the error of gravity.

According to the attitude and velocity error models, it can be concluded that the accuracy of the attitude is related to the velocity while the accuracy of the velocity is related to the specific force measured by the accelerometer. With optimization of the accelerometer, both attitude and velocity errors can be reduced. Therefore, it is effective to optimize the INS by enhancing the performance of the accelerometer.

The output current equation of the accelerometer can be derived as [11]

(5) $$I_{out}=K_{I}\left(a_{i}+a+\frac{1}{K_{B}}\left(M_{d}+K_{h}\beta +\frac{K_{h}\Delta U_{s}}{K_{s}K_{a}}+\frac{K_{h}\Delta U_{a}}{K_{s}}\right)\right)=K_{I}a_{out}$$

where $K_{I}$ is the scale factor between the output current and acceleration $a_{out}$, $a_{i}$ is the input acceleration, a is the cross-coupling acceleration, $K_{B}$ is the accelerometer pendulosity, $M_{d}$ is the disturbance moment, $K_{h}$ is the stiffness of the flexible beam, β is the elastic recovery angle, $K_{s}$ is the signal sensor gain, $\Delta U_{s}$ is the input interference voltage of the servo amplifier, $K_{a}$ is the servo amplifier gain, $\Delta U_{a}$ is the zero drift of the zero amplifier, $K_{a}$ is the scale factor of torque, and I is the output current.

From Equation (5) it can be calculated that the measuring errors of the accelerometer mainly comes from three parts: the error of the scale factor $K_{I}$, the cross-coupling error caused by a and bias errors $\frac{1}{K_{B}}\left(M_{d}+K_{h}\beta +\frac{K_{h}\Delta U_{s}}{K_{s}K_{a}}+\frac{K_{h}\Delta U_{a}}{K_{s}}\right)$ caused by different torques. All of these errors can be influenced by temperature variation and are supposed to be kept at thermal stable conditions. To reduce thermal influences, theoretical analysis proved that the accuracy of accelerometer is able to reach 10^−5^ m/s^2^ when the temperature variation is sustained at 0.01 °C [15].

### 2.2. Verification on Theoretical Analysis Based on BP-NN Algorithm

However, previous results are obtained by approximate calculation and cannot be applied to different INSs directly. Therefore the Back Propagation Neural Network (BP-NN) algorithm is adopted to verify this conclusion. BP-NN is a machine learning algorithm that is able to process the given information to produce an answer. The feasibility of the answer depends on how the internal structure are modified during the learning process. A well-trained BP-NN model is considered to be suitable for solving highly non-linear problems [23,24,25].

Since the algorithm is appropriate for simulation of the nonlinear system, operating temperature and output data of the accelerometer from a specific INS is adopted to build a simulated model. This model is obtained to validate the previous thermal analysis and provide guidelines for precise temperature control system design.

The BP-NN is a multi-layer feedforward network based on the error back propagation algorithm. It can be used to learn relations of the input–output model and it does not need to figure out the mathematical equations that describe these relations. The algorithm contains two processes: the forward propagation of operating signal and theback propagation of the error signal. During back propagation, the weight values of the network are regulated by the error feedback. After continuous modification of weight values, outputs of the network become closer to the expected results. 

**Figure 1:**
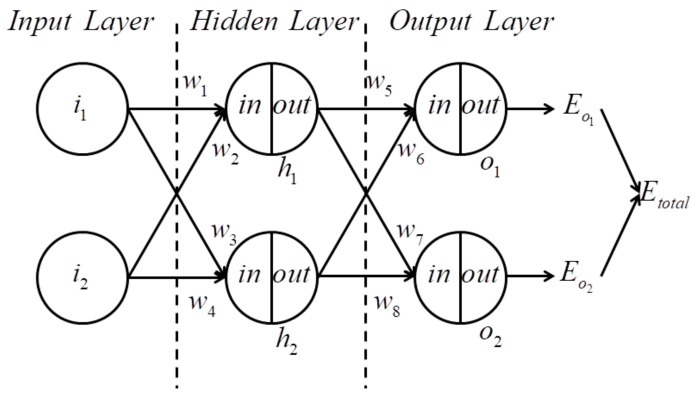
The profile of the three layer BP-NN.

The profile of the three layer BP-NN is shown in Figure 1.

Where i is the input of the Input Layer, $in_{h_{i}},out_{h_{i}}$ are the input and output of the Hidden Layer, respectively, $in_{o_{i}},out_{o_{i}}$ are the input and output of Output Layer, respectively, $w_{i}$ is the weight value of the network, and $E_{o_{i}},E_{total}$ are the output errors of the network.

Assuming that $f\left(x\right)$ is the active function of each layer, then the outputs of the Hidden Layer through forward propagation are

(6) $$\left\{\begin{array}{c} in_{h_{1}}=w_{1}\times i_{1}+w_{2}\times i_{2}\\ in_{h_{2}}=w_{3}\times i_{1}+w_{4}\times i_{2} \end{array}\right.\Rightarrow \left\{\begin{array}{c} out_{h_{1}}=f(in_{h_{1}})\\ out_{h_{2}}=f(in_{h_{2}}) \end{array}\right.$$

The outputs of the Output Layer is

(7) $$\begin{array}{l} \left\{\begin{array}{c} in_{o_{1}}=w_{5}\times out_{h_{1}}+w_{6}\times out_{h_{1}}\\ in_{o_{2}}=w_{7}\times out_{h_{2}}+w_{8}\times out_{h_{2}} \end{array}\right.\\ \Rightarrow \left\{\begin{array}{c} out_{o_{1}}=f(in_{o_{1}})\\ out_{o_{2}}=f(in_{o_{2}}) \end{array}\right. \end{array}$$

Errors of the output are

(8) $$\left\{\begin{array}{c} E_{o_{1}}=\frac{1}{2}{\left(o_{1}-out_{o_{1}}\right)}^{2}\\ E_{o_{2}}=\frac{1}{2}{\left(o_{2}-out_{o_{2}}\right)}^{2}\\ E_{total}=E_{o_{1}}+E_{o_{2}} \end{array}\right.$$

where $o_{i}$ is the expected output of different propagation paths.

After forward propagation, the weight value of the network should be regulated through back propagation. Take $w_{5}$ as an example, the error function of the Output Layer is shown below

(9) $$\frac{\partial E_{total}}{\partial w_{5}}=\frac{\partial E_{total}}{\partial out_{o_{1}}}\frac{\partial out_{o_{1}}}{\partial in_{o_{1}}}\frac{\partial in_{o_{1}}}{\partial w_{5}}$$

Assuming that $w_{5}^{+}$ is the regulated weight value, it can be derived as

(10) $$w_{5}^{+}=w_{5}-\eta \times \frac{\partial E_{total}}{\partial w_{5}}$$

In this formula, η represents the learning rate and the regulated weight values of $w_{6}$, $w_{7}$, $w_{8}$ can be generated in a similar manner..

Take $w_{1}$ as an example, the error function of the Hidden Layer is expressed below

(11) $$\frac{\partial E_{total}}{\partial w_{1}}=\frac{\partial E_{total}}{\partial out_{h_{1}}}\frac{\partial out_{h_{1}}}{\partial in_{h_{1}}}\frac{\partial in_{h_{1}}}{\partial w_{1}}\;=\left(\frac{\partial E_{o_{1}}}{\partial out_{h_{1}}}+\frac{\partial E_{o_{2}}}{\partial out_{h_{1}}}\right)\frac{\partial out_{h_{1}}}{\partial in_{h_{1}}}\frac{\partial in_{h_{1}}}{\partial w_{1}}\;=\left(\begin{array}{c} \frac{\partial E_{o_{1}}}{\partial out_{o_{1}}}\frac{\partial out_{o_{1}}}{\partial in_{o_{1}}}\frac{\partial in_{o_{1}}}{\partial out_{h_{1}}}+\\ \frac{\partial E_{o_{2}}}{\partial out_{o_{2}}}\frac{\partial out_{o_{2}}}{\partial in_{o_{2}}}\frac{\partial in_{o_{2}}}{\partial out_{h_{1}}} \end{array}\right)\frac{\partial out_{h_{1}}}{\partial in_{h_{1}}}\frac{\partial in_{h_{1}}}{\partial w_{1}}$$

Assuming that $w_{1}^{+}$ is the regulated weight value, it can be derived as

(12) $$w_{1}^{+}=w_{1}-\eta \times \frac{\partial E_{total}}{\partial w_{1}}$$

and $w_{2}$, $w_{3}$, $w_{4}$ can be generated in a similar manner. Repeating forward and back propagation calculations, the BP-NN model can gradually determine the relations between input and output data. 

**Figure 2:**
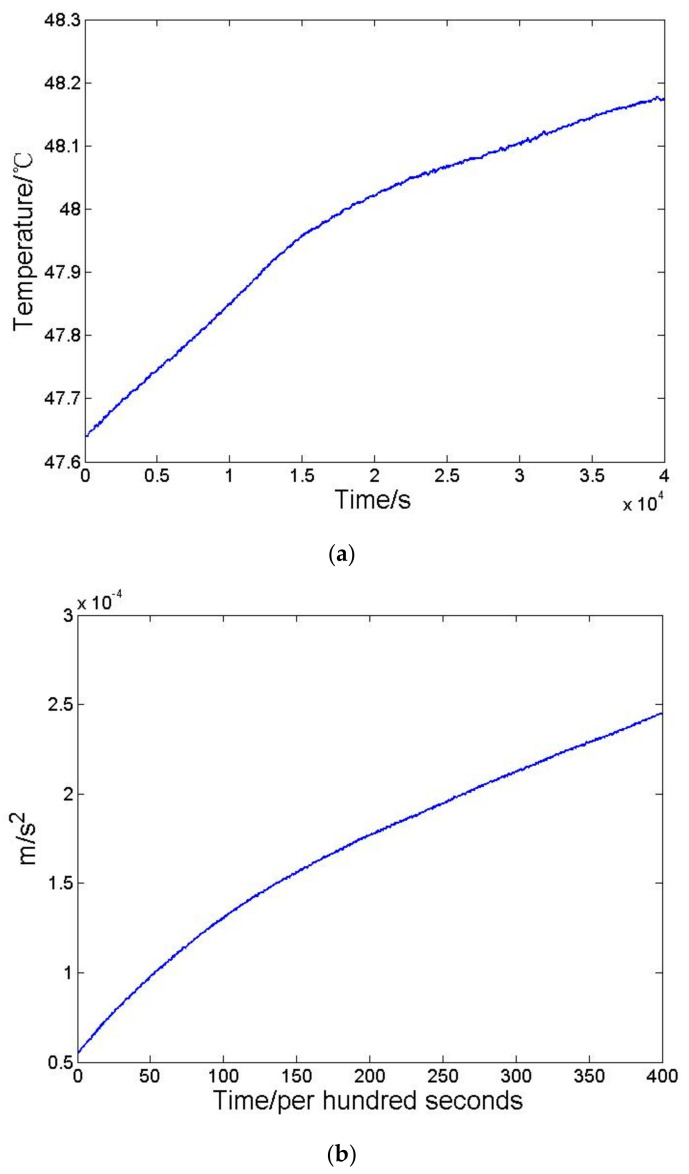
(**a**) The temperature variation of the accelerometer, and (**b**) the mean value per hundred seconds of accelerometer outputs.

In order to verify the thermal conclusion, a three layer BP-NN is chosen and the sigmoid function is selected as the active function. The input data of the first layer is the operating temperature variation of the accelerometer, and the output data of the third layer is the mean value per hundred seconds of acceleration measured by the accelerometer. Through back and forth propagation processes, weight values of each layer are continuously modified. Finally, the relation model between operating temperature and acceleration of the accelerometer can be obtained. The input and output data are shown in Figure 2.

The standard deviation of temperature and mean square deviation per hundred seconds of accelerometer outputs are shown in Table 1.

**Figure 3:**
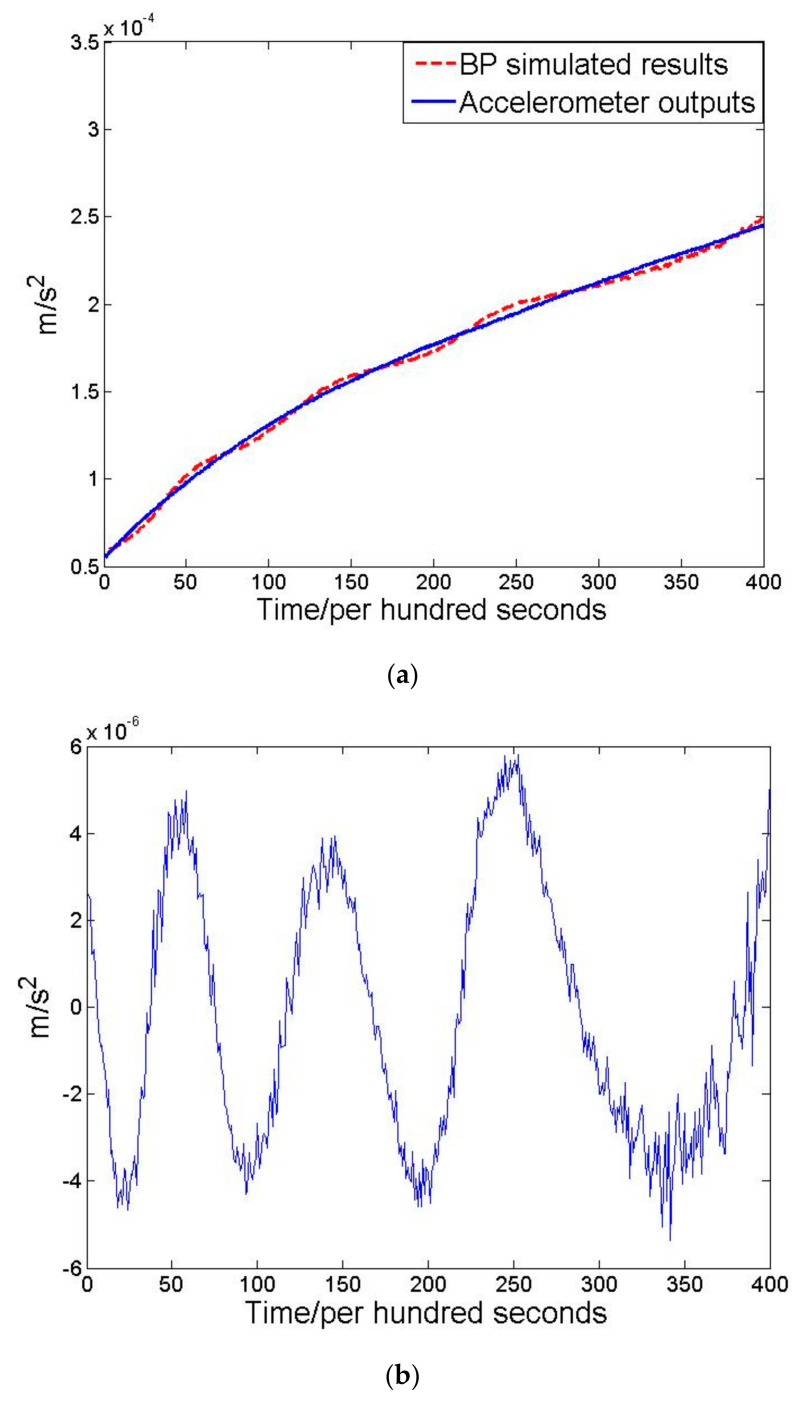
(**a**) The mean value per hundred seconds of accelerometer outputs and (**b**) the mean value per hundred seconds of accelerometer outputs.

After BP-NN simulation, the mean value per hundred seconds of simulated results and actual outputs are shown in Figure 3a. Errors are shown in Figure 3b.

From the error results it can be concluded that the BP-NN model has good prediction between input and output data while errors are less than 10^−5^ m/s^2^. Based on this well-trained model, different temperature data are chosen to verify theoretical thermal analysis. The test results are shown in Table 2.

Theoretical analysis points out that when temperature variation is less than 0.01 °C, the accuracy of the accelerometer can be sustained at 10^−5^ m/s^2^. After BP-NN simulation, the test results show that a 0.01 °C temperature variation leads to 0.9339 × 10^−5^ m/s^2^ output accuracy. This conclusion proves the theoretical analysis and provides guidelines for precise temperature control system design. 

### 2.3. Profile of Precise Temperature Control System

**Figure 4:**
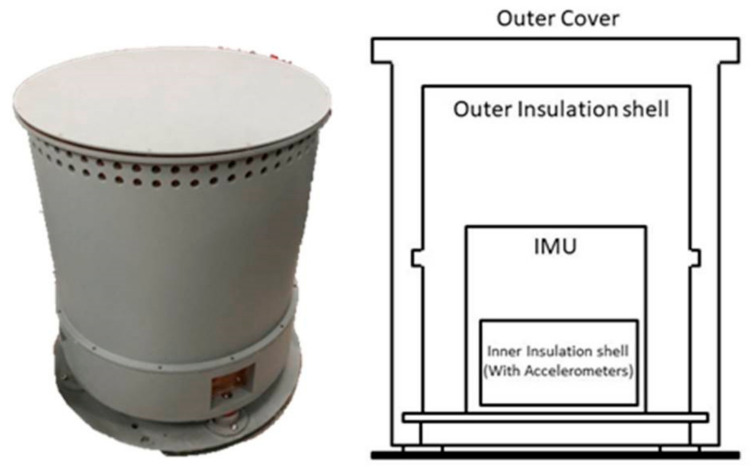
The profile of the INS with precise temperature control system.

As mentioned above, the accelerometer accuracy can reach 10^−5^ m/s^2^ when its operating temperature variation is less than 0.01 °C. Thus, in order to improve the output accuracy, a multi-layer precise temperature control scheme is adopted [17]. The profile of the INS with precise temperature control system is shown in Figure 4.

Based on previous studies, the multi-level control system is more effective in temperature isolation than the single-level system. Therefore, multi-level structure design is adopted to build up the precise temperature control system. 

Extra outer and inner insulation shells are added into the control system as the first and second thermal isolation layers. Environment temperature variation can be diminished and the inner temperature of the RLG INS can also be maintained in stable condition through these shells.

In addition, PID control circuits with TEC (cooling components) and PTC (heating components) are also combined together in order to enhance the temperature control performance under different conditions. Both the TEC and PTC components can actively change the inner temperature of the insulation shells. With passive thermal control from insulation shells and active thermal control from TEC/PTC, the operating temperature variation of the accelerometer can be steadily sustained at 0.01 °C.

## 3. Results

After building up the multi-level temperature control system, experiments were carefully designed to verify its feasibility. Contrast experiments were conducted to test the control capability of the system, then performance experiments were designed to observe how the system operates under different conditions. Finally attitude and static navigation experiments were set up to validate system measurement improvements on the RLG INS. 

### 3.1. Contrast Experiments on Precise Temperature Control System

**Figure 5:**
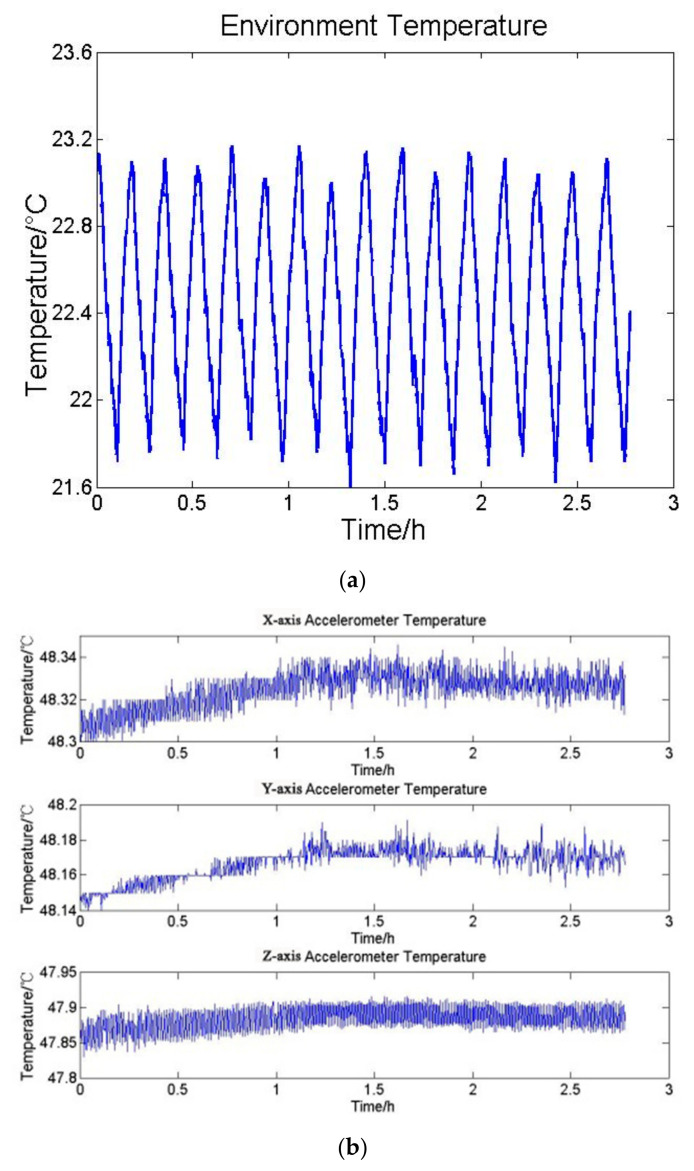

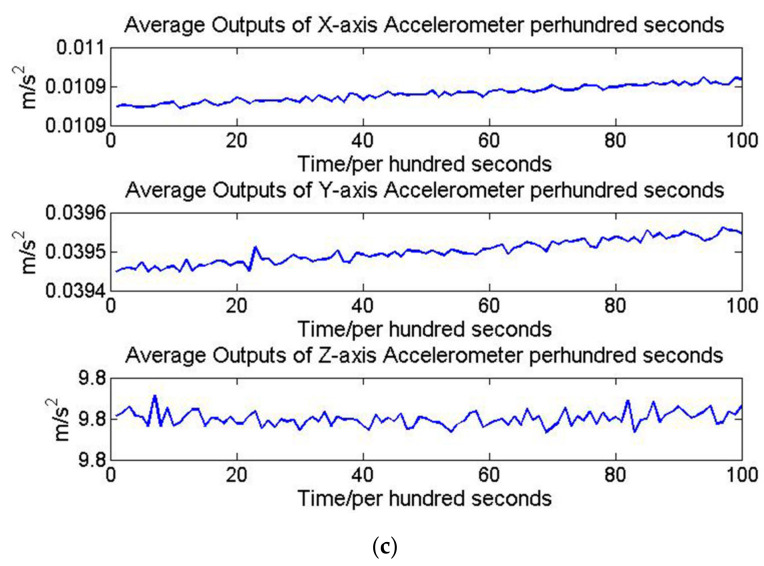
(**a**) Environment temperature variation, (**b**) accelerometers temperature variation, and (**c**) the average outputs of accelerometers per hundred seconds.

At first the INS stays still and is tested under the laboratory environment without the temperature control system. The duration of the experiment is 3 h. The environment temperature variation, accelerometers temperature variation and average output of accelerometers per hundred seconds are shown in Figure 5.

In this test the standard deviation of environment temperature variation is 0.4658 °C. The standard deviation of the accelerometers temperature variations and outputs are shown in Table 3. The temperature deviations of three accelerometers are around 0.05 °C and output accuracy deviations are also larger than 5 × 10^−5^ m/s^2^.

**Figure 6:**
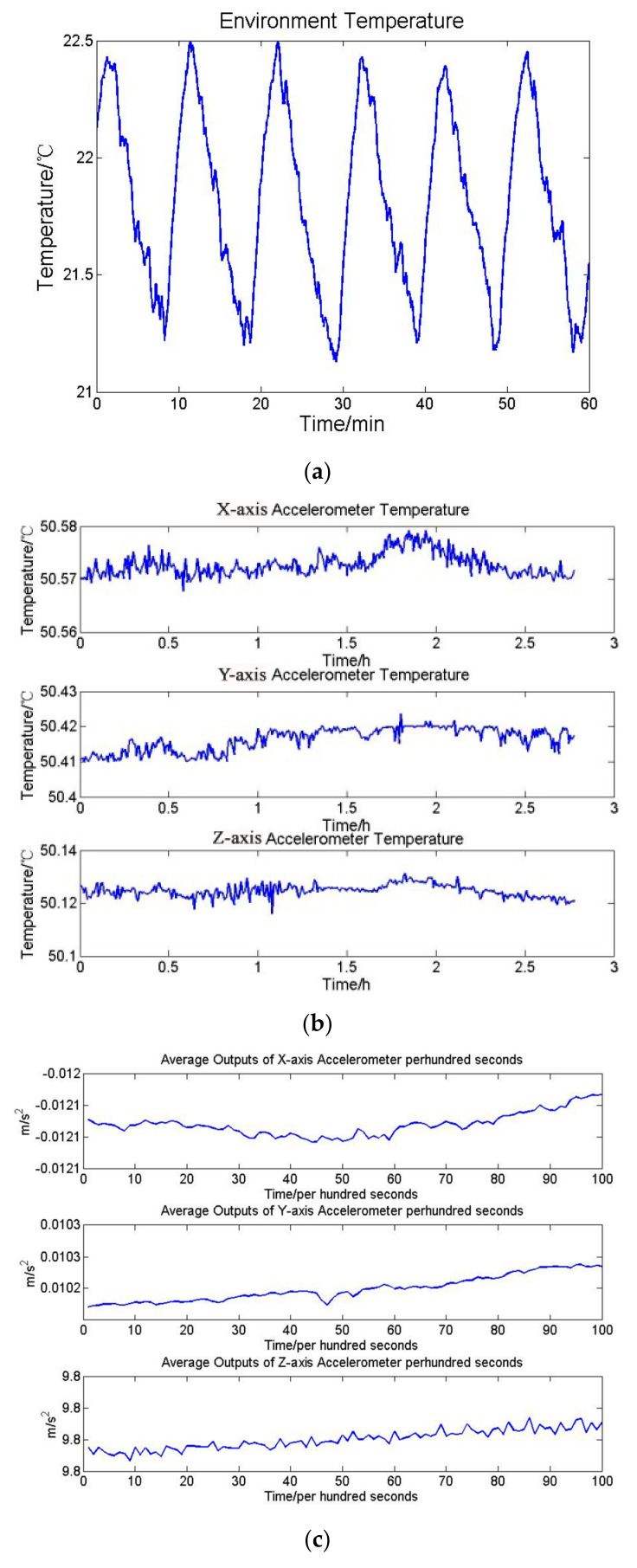
(**a**) Environment temperature variation (**b**) accelerometers temperature variation and (**c**) the average outputs of accelerometers per hundred seconds.

The INS was then tested under laboratory environment conditions with the temperature control system turning on. Meanwhile, other experiment conditions remained unchanged. The environment temperature variation, accelerometers temperature variation and average output of accelerometers per hundred seconds are shown in Figure 6.

In this test the standard deviation of environment temperature variation is 0.3714 °C. The standard deviation of the accelerometers temperature variations and outputs are shown in Table 4.

According to the experiment results, temperature deviations of all three accelerometers are able to remain less than 0.01 °C. In addition, output accuracy deviations of the three accelerometers are also less than 1 × 10^−5^ m/s^2^, as planned.

### 3.2. Performance Experiments on Precise Temperature Control System

**Figure 7:**
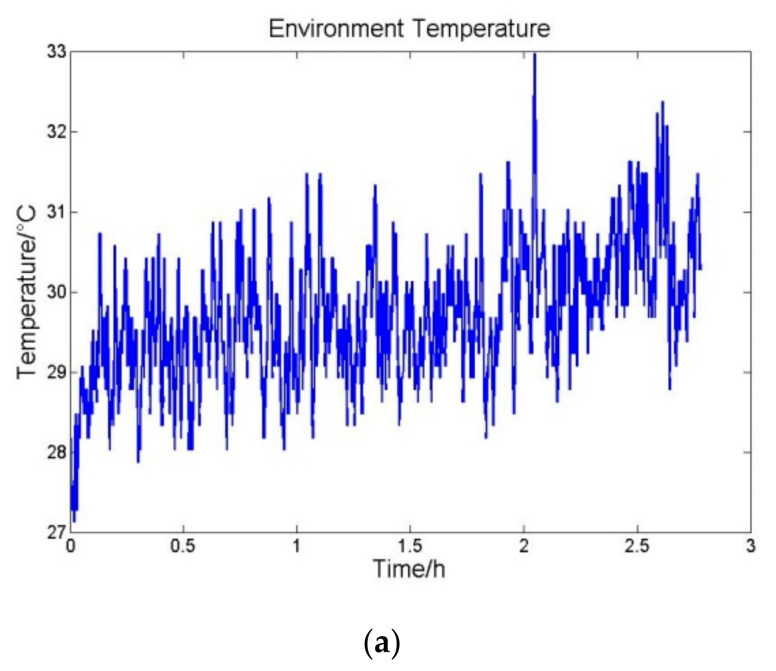

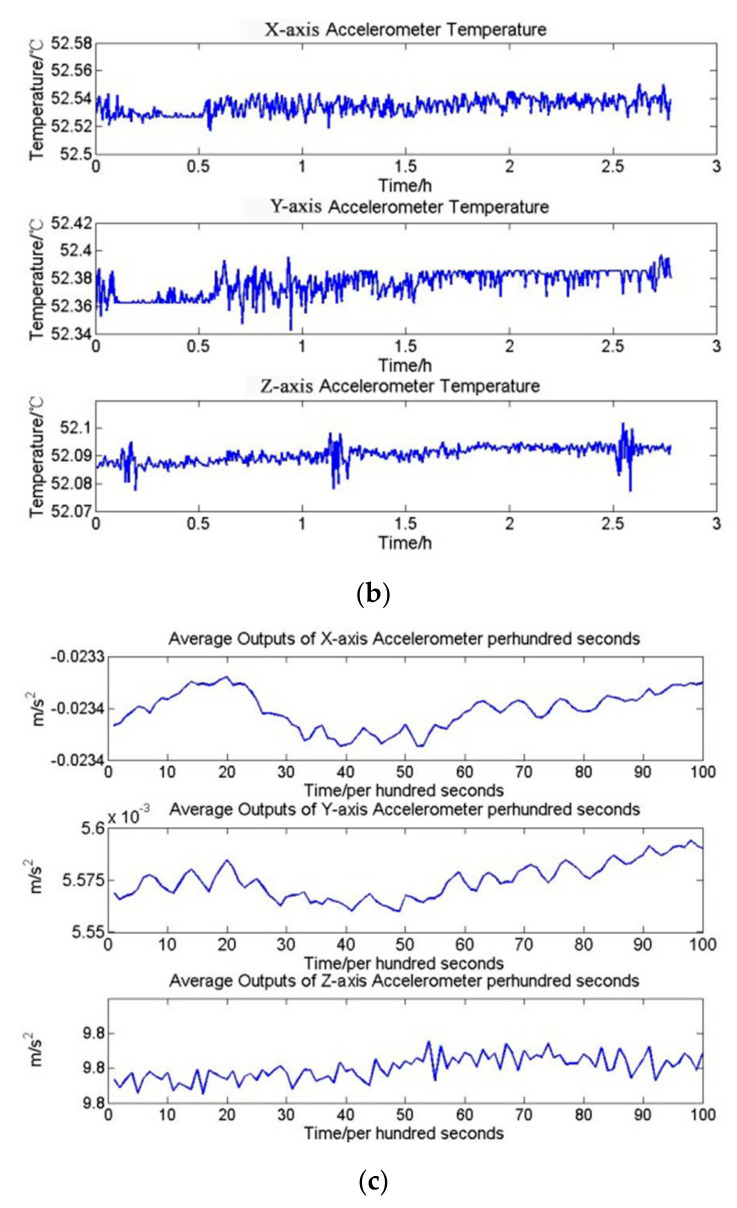
(**a**) Environment temperature variation, (**b**) accelerometers temperature variation and (**c**) the average outputs of accelerometers per hundred seconds.

**Figure 8:**
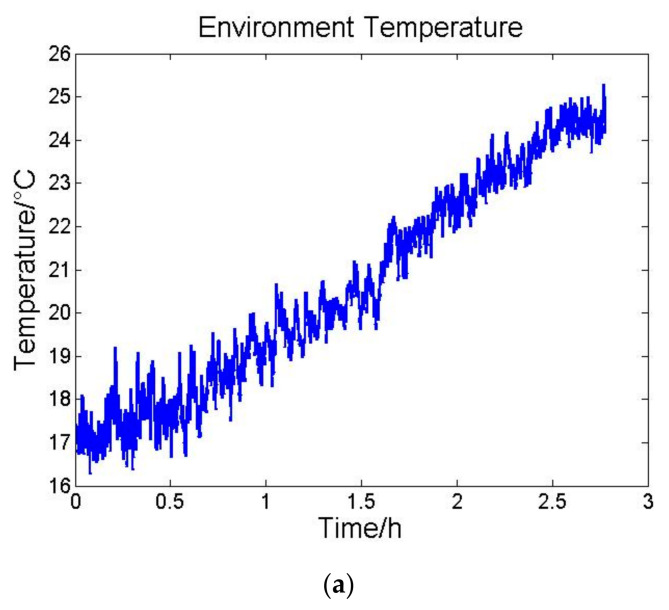

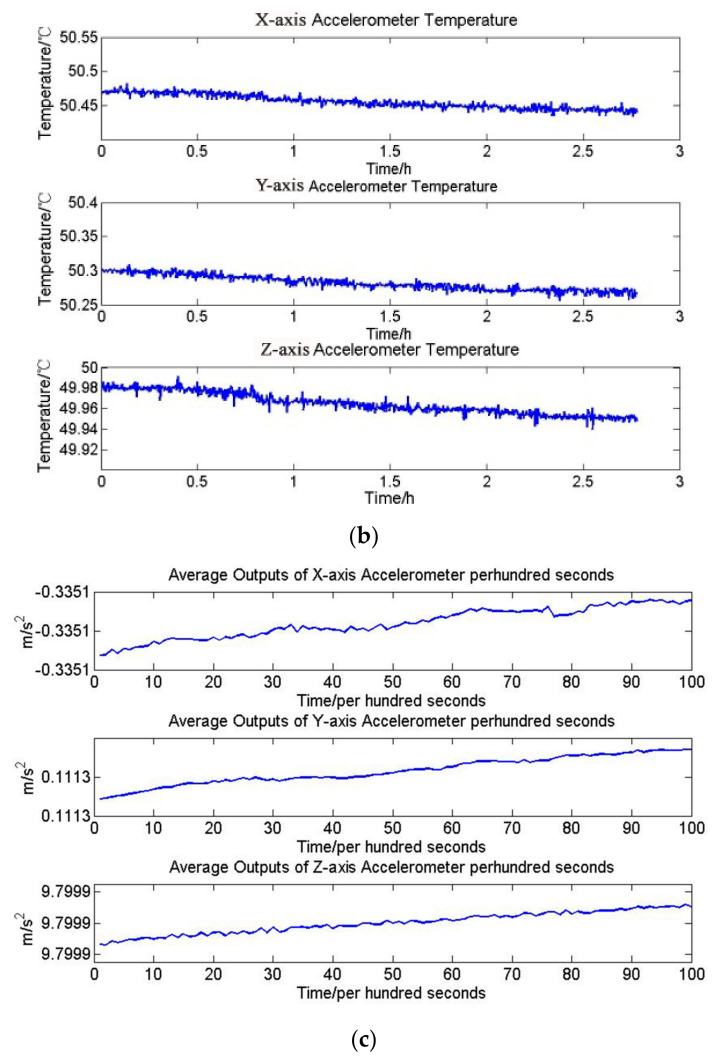
(**a**) Environment temperature variation, (**b**) accelerometers temperature variation, and (**c**) the average outputs of accelerometers per hundred seconds.

After laboratory tests, the INS with precise temperature control system was operating under different conditions to evaluate thermal performance. The whole system was tested in an experimental vehicle and a temperature control incubator separately. Experiment duration was still 3 h. The vehicle experiment and temperature control incubator experiment results are shown in Figure 7 and Figure 8, respectively.

In the vehicle test the standard deviation of environment temperature variation is 0.8067 °C, and environment temperature standard deviation of the temperature control incubator is 2.4662 °C.

The standard deviation results of the two experiments are shown in Table 5 and Table 6.

### 3.3. Attitude and Static Navigation Experiments 

**Figure 9:**
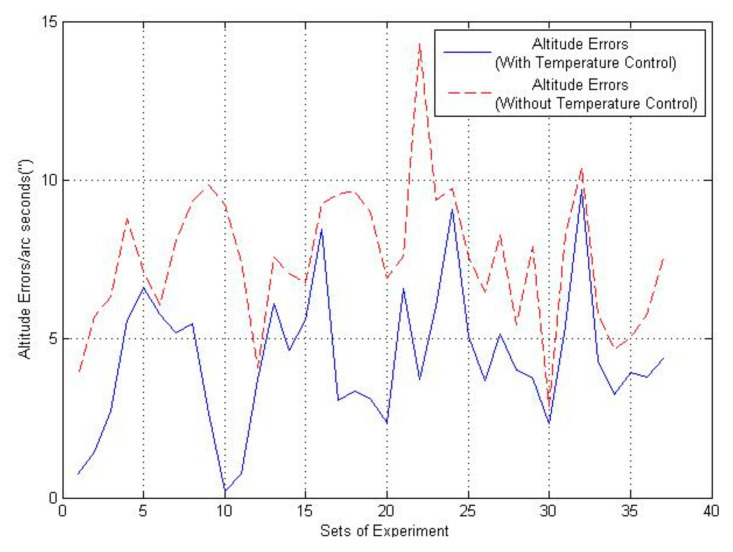
Altitude errors.

In order to further validate the performance of the precise temperature control system, the attitude test and static navigation test have been designed. In the attitude test, the INS is fixed in a turntable with and without the precise temperature control system. The RLG INS rotates around the z-axis by turntable at a given angle and then stays still. Thirty-six sets of data were obtained and analyzed through experiments. The attitude errors of yaw angular are shown in Figure 9 and Table 7. From attitude experiments, the maximum, minimum and mean values of attitude errors of the RLG INS with temperature control are 0.75″, 9.69″ and 4.37″, respectively, while those of the RLG INS without temperature control are 2.88″, 14.30″ and 7.53″, respectively. Additionally, the mean attitude error has been decreased by 41.97% after temperature control and validates the temperature control system in altitude calculation.

**Figure 10:**
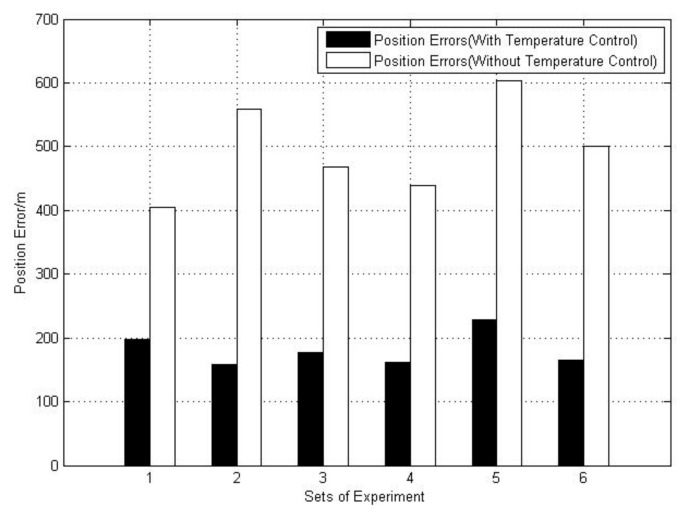
Position errors.

In the static navigation test, the INS is fixed in a platform with and without the precise temperature control system. The experiment duration is 1 h and 6 sets of data were analyzed. The position errors are shown in Figure 10 and Table 8. In the static navigation tests, the mean values of position errors of the RLG INS with temperature control are 181.44 m, while those of the RLG INS without temperature control are 491.64 m. Thus, the mean position errors were decreased by 62.91%. According to comparison results, it can be easily concluded that the precision temperature control system can undoubtedly improve the performance of the INS by over 60%.

## 4. Discussion

This section discusses results generated from the experiments above and validates the feasibility of the precise temperature control system.

In the contrast experiment, it can be concluded that without the precise temperature control system, the operating temperature variation of the accelerometers cannot sustain a variation of 0.01 °C, and output accuracy cannot be kept at less than 1 × 10^−5^ m/s^2^. Additionaly, this result also proves the necessity of the precise temperature control system.

The results of the temperature controlled INS not only verify the theoretical analysis and BP-NN simulation conclusions, but also validate the performance of the precise temperature control system. Additionally, it can be concluded that the Z-axis accelerometer is the most thermally insensitive inertial sensor while Y-axis one is the worst among the three sensors. 

In the performance experiments, it can be concluded that even when the environment temperature varies rapidly in the vehicle and temperature control incubator, the control system can still keep the accelerometer operating temperature at a proper point, as designed. As the temperature variation increasing, the output accuracy deviations also rises, but still remain less than 1 × 10^−5^ m/s^2^. Therefore, the precise temperature control system was verified to be effective in different temperature conditions through performance experiments.

In the attitude experiment, it can be seen that attitude errors of the INS with temperature control is relatively smaller than that of INS without temperature control. Even though the attitude errors change in different experiments, results of the thermal stable system are commonly better than the system influenced by temperature variations. 

In the static navigation tests, the east and north position errors of the INS with the temperature control system are also less than that of the INS without the temperature control system by over 100 m. These results agree with BP-NN conclusions and validate the effectiveness of the multi-level temperature control system.

According to the experiments above, the precise temperature control system proves to be useful comparing with the INS without temperature control. The system is able to maintain the temperature variation of the accelerometer at 0.01 °C and make the output accuracy match up with requirements at 1 × 10^−5^ m/s^2^. Theoretical thermal analysis has been validated and the precise temperature control system can be effective in different temperature conditions. Additionally, attitude and position accuracy can also be improved through precise temperature control.

## 5. Conclusions

This article discusses the thermal influences on the accelerometer and the INS. Theoretical analysis has been made and BP-NN algorithm is adopted to build simulated models of the accelerometer and verify the temperature control conclusion. After that, a multi-layer temperature control system was set up to further support the verification of the BP-NN simulation. Experiments were carefully designed and the system was tested under different operating conditions. The results of the contrast and performance experiments verify the effectiveness of the control system. With precise temperature control, the operating temperature of the accelerometer was able to be sustained at 0.01 °C and its output accuracy reached 1 × 10^−5^ m/s^2^. Based on the precision temperature control system, static altitude and navigation results were improved by 41.97% and 62.91%, respectively, in typical experiment conditions, which not only advances the performance of the INS, but also makes it possible to measure the gravity anomaly. With further study, measurement accuracy of the INS can be enhanced through gravity anomaly aided navigation in long-endurance marine navigation and underwater navigation. 

## Figures and Tables

**Table 1:** The standard deviation of temperature and the mean square deviation per hundred seconds of accelerometer.

Time (s)	Standard Deviation of Temperature (°C)	Mean Square Deviation Per Hundred Seconds of Accelerometer Outputs (×10^−5^ m/s^2^)
1–10,000	0.0602	13.2301
10,001–20,000	0.0513	10.3651
20,001–30,000	0.0227	3.7755
30,001–40,000	0.0216	3.2664

**Table 2:** Test results of different temperature variation.

Time(s)	Standard Deviation of Temperature (°C)	Mean Square Deviation Per Hundred Seconds of Accelerometer Outputs (×10^−5^ m/s^2^)
1–10,000	0.02	3.1290
10,001–20,000	0.015	1.7060
20,001–30,000	0.01	0.9339
30,001–40,000	0.005	0.5892

**Table 3:** The deviation of accelerometer temperature and mean square deviation per hundred seconds of accelerometer outputs.

	*X*-Axis Accelerometer	*Y*-Axis Accelerometer	*Z*-Axis Accelerometer
Deviation of Temperature	0.0568 °C	0.0542 °C	0.0503 °C
Mean square deviation per hundred seconds of Outputs	5.2985 × 10^−5^ m/s^2^	5.9909 × 10^−5^ m/s^2^	5.0517 × 10^−5^ m/s^2^

**Table 4:** The deviation of accelerometer temperature and mean square deviation per hundred seconds of accelerometer utputs.

	*X*-Axis Accelerometer	*Y*-Axis Accelerometer	*Z*-Axis Accelerometer
Deviation of Temperature	0.0074 °C	0.0080 °C	0.0067 °C
Mean square deviation per hundred seconds of Outputs	0.7306 × 10^−5^ m/s^2^	0.8045 × 10^−5^ m/s^2^	0.6151 × 10^−5^ m/s^2^

**Table 5:** The deviation of accelerometer temperature and mean square deviation per hundred seconds of accelerometer outputs in vehicle test.

	*X*-Axis Accelerometer	*Y*-Axis Accelerometer	*Z*-Axis Accelerometer
Deviation of Temperature	0.0079 °C	0.0085 °C	0.0070 °C
Mean square deviation per hundred seconds of Outputs	0.7807 × 10^−5^ m/s^2^	0.8758 × 10^−5^ m/s^2^	0.6931 × 10^−5^ m/s^2^

**Table 6:** The deviation of accelerometer temperature and mean square deviation per hundred seconds of accelerometer outputs in temperature control incubator test.

	*X*-Axis Accelerometer	*Y*-Axis Accelerometer	*Z*-Axis Accelerometer
Deviation of Temperature	0.0087 °C	0.0091 °C	0.0081 °C
Mean square deviation per hundred seconds of Outputs	0.8026 × 10^−5^ m/s^2^	0.9190 × 10^−5^ m/s^2^	0.7480 × 10^−5^ m/s^2^

**Table 7:** Altitude errors of yaw angular.

Sets of Experiment	Altitude Error/ Arc Seconds (With Temperature Control)	Altitude Error/ Arc Seconds (Without Temperature Control)	Percetange Improvements
1	0.75	3.83	80.41%
2	1.45	5.69	74.51%
3	2.74	6.33	56.71%
4	5.59	8.79	36.40%
5	6.63	7.14	7.14%
6	5.76	6.07	5.10%
7	5.19	8.07	35.68%
8	5.50	9.32	40.98%
9	2.66	9.83	72.93%
10	0.21	9.22	97.72%
11	0.74	7.43	90.04%
12	3.74	4.08	8.33%
13	6.11	7.59	19.49%
14	4.64	7.07	34.37%
15	5.59	6.75	17.18%
16	8.45	9.27	8.84%
17	3.08	9.56	67.78%
18	3.35	9.65	65.28%
19	3.11	8.95	65.25%
20	2.38	6.93	65.65%
21	6.58	7.62	13.64%
22	3.71	14.30	74.05%
23	6.02	9.36	35.68%
24	9.06	9.72	6.79%
25	5.08	7.56	32.80%
26	3.69	6.48	43.05%
27	5.15	8.28	37.80%
28	4.00	5.43	26.33%
29	3.76	7.92	52.52%
30	2.34	2.88	18.75%
31	5.41	8.28	34.66%
32	9.69	10.4	6.82%
33	4.32	5.76	25%
34	3.24	4.68	30.76%
35	3.96	5.04	21.42%
36	3.78	5.76	34.37%
Mean Value	4.37	7.53	41.97%

**Table 8:** Position errors of the static navigation.

Sets of Experiments	Position Error/m (With Temperature Control)	Position Error/m (Without Temperature Control)	Percentage Improvement
1	196.87	404.82	51.36%
2	158.35	559.42	71.69%
3	177.00	468.58	62.22%
4	161.89	439.61	63.17%
5	228.87	603.10	62.051%
6	165.68	501.40	66.95%
Mean Value	181.44	496.16	62.91%

## Data Availability

The data presented in this study are available on request from the corresponding author. The data are not publicly available due to privacy reasons.
